# Hypermasculinised facial morphology in boys and girls with Autism Spectrum Disorder and its association with symptomatology

**DOI:** 10.1038/s41598-017-09939-y

**Published:** 2017-08-24

**Authors:** Diana Weiting Tan, Syed Zulqarnain Gilani, Murray T. Maybery, Ajmal Mian, Anna Hunt, Mark Walters, Andrew J. O. Whitehouse

**Affiliations:** 1 Neurocognitive Developmental Unit, School of Psychological Science, University of Western Australia, Perth, 6009 Western Australia Australia; 2 Telethon Kids Institute, University of Western Australia, Perth, 6008 Western Australia Australia; 3 School of Computer Science and Software Engineering, University of Western Australia, Perth, 6009 Western Australia Australia; 4 Craniomaxillofacial Department, Princess Margaret Hospital for Children, Perth, 6008 Western Australia Australia

## Abstract

Elevated prenatal testosterone exposure has been associated with Autism Spectrum Disorder (ASD) and facial masculinity. By employing three-dimensional (3D) photogrammetry, the current study investigated whether prepubescent boys and girls with ASD present increased facial masculinity compared to typically-developing controls. There were two phases to this research. 3D facial images were obtained from a normative sample of 48 boys and 53 girls (3.01–12.44 years old) to determine typical facial masculinity/femininity. The sexually dimorphic features were used to create a continuous ‘gender score’, indexing degree of facial masculinity. Gender scores based on 3D facial images were then compared for 54 autistic and 54 control boys (3.01–12.52 years old), and also for 20 autistic and 60 control girls (4.24–11.78 years). For each sex, increased facial masculinity was observed in the ASD group relative to control group. Further analyses revealed that increased facial masculinity in the ASD group correlated with more social-communication difficulties based on the Social Affect score derived from the Autism Diagnostic Observation Scale-Generic (ADOS-G). There was no association between facial masculinity and the derived Restricted and Repetitive Behaviours score. This is the first study demonstrating facial hypermasculinisation in ASD and its relationship to social-communication difficulties in prepubescent children.

## Introduction

Autism Spectrum Disorder (ASD) is a neurodevelopmental condition characterised by difficulties in social communication and the presence of restricted interests and repetitive behaviours^1^. While genetic factors are known to play a major role in ASD^2–4^, a growing body of scientific literature has examined the influence of endocrine factors in the development of the condition. The ‘extreme male brain’ theory holds that cognitive and behavioural characteristics of ASD represent an extreme form of the typical male phenotype as a result of exposure to elevated levels of prenatal testosterone^5^. However, empirical findings accumulated from the past few decades of research suggest that the link between prenatal testosterone and behaviours associated with ASD is not a clear-cut one.

Whilst several studies reported that increased concentrations of prenatal testosterone were related to more pronounced autistic traits^6, 7^, increased male-type play^8^, enhanced visuospatial skills^9^, and poorer social and language skills^10^, other studies have either identified a link restricted to one sex^11–13^ or observed no association in both sexes^14–17^. Also, a recent study of typically-developing children with an older sibling diagnosed with ASD^18^ found that elevated levels of prenatal testosterone were not associated with autistic traits in the full sample, but an association was found for the subgroup of children who had a female older sibling with ASD. Furthermore, a case-control study of boys with and without ASD^19^ indicated that whereas the groups had comparable prenatal testosterone concentrations, a latent factor score derived from prenatal levels of cortisol and steroids associated with the biosynthesis of testosterone showed elevated levels in the ASD group compared to the controls. These inconsistent results may be attributed to methodological differences in deriving prenatal testosterone measures (i.e., the use of amniotic fluid or umbilical cord blood^20^) and possible influences of environmental factors^21^ such as parenting styles^22^ on autistic traits.

A parallel stream of research found that heightened prenatal testosterone levels are related to facial morphology. In a twin study, Marečková *et al*.^23^ found that eight-year-old girls with a male co-twin, presumably exposed to higher testosterone levels *in utero*, showed less feminine skeletal craniofacial structures than girls with a female co-twin. Using a more direct approach, Whitehouse *et al*.^24^ identified a positive relationship between prenatal testosterone measured from umbilical cord blood and hypermasculinised facial phenotypes in early adulthood when neurotypical men and women were analysed together and separately. Taken together, evidence linking prenatal testosterone exposure to both ASD and facial hypermasculinisation leads to an hypothesis that individuals with ASD may also present with hypermasculinised facial phenotypes^25–27^.

A number of studies have examined the hypermasculinisation account in neurotypical adults selected for high and low levels of autistic-like traits, revealing inconsistent findings. Scott *et al*.^27^ found that composite images created from groups of men with high levels of autistic-like traits were subjectively rated as being more masculine than images created from low-trait men. However, this effect was absent in female samples. In contrast, Tan *et al*.^25^ did not observe a difference in facial masculinity between men with high and low levels of autistic-like traits, but found that women with high trait levels were rated as being less feminine than women with low levels of autistic-like traits. Gilani *et al*.^26^ extended this work by assessing facial masculinity using objective measurements of sexually dimorphic facial features taken from three-dimensional (3D) facial images. Less feminine facial features were reported in women with high levels of autistic-like traits compared to women with low trait levels. However, features of men with high trait levels were found to be less masculine than those of men with low trait levels.

Another study has examined the extent of gender coherence in the physical morphology (e.g., faces, voices and bodies) of adults diagnosed with ASD. Consistent with the previous findings in neurotypical women, Bejerot *et al*.^28^ found that faces of autistic women were subjectively rated as being less gender typical than the faces of non-autistic women. Although faces of autistic men were not rated differently to those of male controls, bodies and voices of men with ASD were perceived as less gender typical than these features of non-autistic men. Bejerot *et al*.^28^ proposed that autistic physical features are better described as being androgynous in form whereby females with ASD are less feminised and males with ASD are less masculinised relative to individuals of typical development. This androgyny account is in line with several reports of higher incidence of gender dysphoria in both males and females with ASD compared to the general population^29, 30^.

Research to date suggests a stable pattern of results for female adults whereby a hypermasculinised (or androgynous) facial appearance has been observed in women with subclinical autistic traits and clinical ASD^25, 26, 28^. However, the relationship between facial masculinity and autistic traits is less clear for men, with hypermasculinised features reported in men with more profound autistic-like traits in one study^27^, androgynous features observed in men with subclinical and clinical ASD^26, 28^, and non-significant differences being reported in two further studies^25, 27^. Until now, investigations into the hypermasculinisation and androgyny accounts have been focused on the association between autism and facial morphology in post-pubertal adults. As adult facial morphology may be influenced by pubertal hormone actions^31^, the current study built on the existing evidence by examining facial morphology in prepubescent children with and without ASD.

Preliminary investigations of face morphology have been conducted in boys with and without ASD, employing 3D photogrammetry, which allows for fine-grained, precise and replicable analyses^32^. These studies found that boys with ASD present a distinct set of facial features compared to typically-developing boys, including greater facial asymmetry^33^, decreased facial height and increased breadth of mouth using the linear distance between pairs of facial landmarks (i.e. straight-line distance ‘cutting through’ facial surface)^34^, and increased facial protrusion using geodesic distance (i.e. topographical distance over facial surface)^35^. However, as these studies employed an hypothesis-free approach in their investigations, there remains little understanding of the factor(s) underpinning the facial phenotype associated with ASD. Furthermore, no studies have examined the facial morphology of girls with ASD.

To this end, the current study adopted an hypothesis-driven approach in comparing the facial masculinity/femininity of boys and girls with ASD to that of typically-developing children. This investigation was carried out in two steps. First, we identified a set of facial features that differentiated the faces of typically-developing boys and girls. In the second step, we used this set of sexually dimorphic facial features to compare facial structure of children with and without ASD. If the androgyny account is supported, we would expect less masculine faces in boys with ASD relative to control boys, and less feminine faces in girls with ASD relative to control girls. Conversely, if the hypermasculinisation account is supported, we should observe more pronounced facial masculinisation/defeminisation in both autistic boys and girls compared to non-autistic controls.

## Study 1

We employed the gender classification algorithm described in Gilani *et al*.^36^ to identify a set of facial features that optimally distinguish the faces of prepubescent typically-developing boys and girls. This algorithm was previously used to identify sexually dimorphic facial features of young adult men and women in our previous research^24, 26^.

## Method

### Participants

Typically-developing boys and girls were recruited from local schools, childcare centres and a science discovery centre. None of their parents reported any health or developmental conditions or treatments that may have an impact on facial structure. To minimise the effects of ethnic variability on the facial measurements, samples were restricted to Caucasian children. Samples matched for age of 48 boys (*M* = 7.86 years, *SD* = 3.10, range = 3.01–12.40 years) and 53 girls (*M* = 7.93 years, *SD* = 2.77, range = 3.01–12.44 years) were obtained for further analyses. Ethical approval for the study was obtained from the human research ethics committees at the University of Western Australia and Princess Margaret Hospital for Children, and informed consents to participate were obtained from parents or legal guardians.

### 3D facial photography

Facial images were acquired using a 3dMDface system (3dMD, Atlanta, GA, USA) operated from a desktop computer. The 3dMDface system produces 3D ear-to-ear facial images using random infrared light projection on the face of the subject and combining multiple images captured from colour texture and infrared cameras from two stereo camera viewpoints. The use of infrared projectors and cameras allows the 3D facial shape to be measured to sub-millimetre accuracy. The children sat in front of the 3dMDface system with their gaze fixed on a sticker pasted on a wall behind the machine. They were instructed to maintain a neutral facial expression and keep their lips closed in centric jaw relation during the imaging process.

### Gender classification algorithm

**Figure 1:**
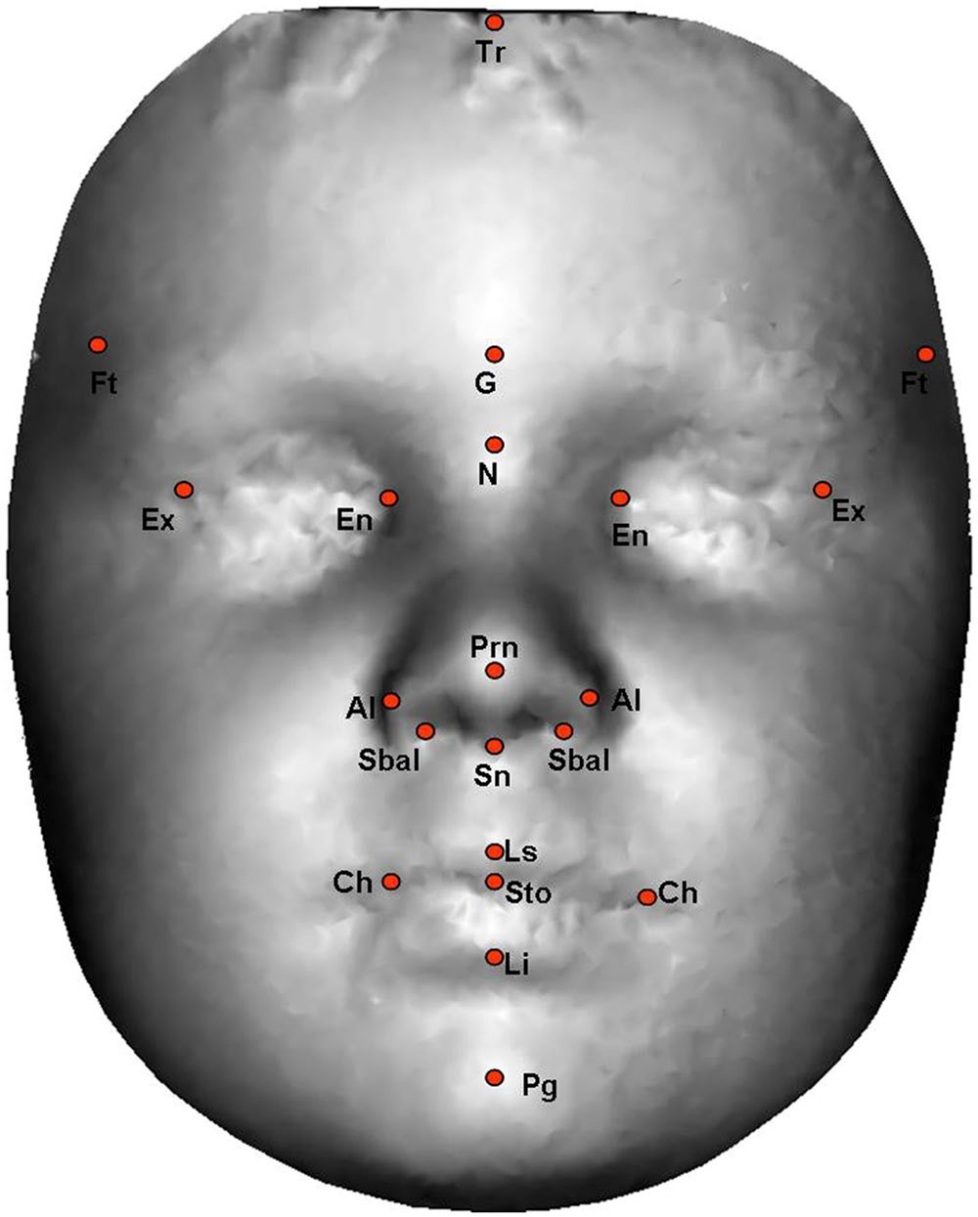
3D image annotated with 21 facial landmarks. These landmarks can be visually identified without manual palpation and images can be manipulated (e.g., rotated and toggled between shaded and coloured textures) in Matlab™ to improve annotation accuracy.

In the first stage of the analysis, 26 linear distances and 26 geodesic distances were measured from 21 landmarks annotated on each 3D facial image^37^ (see Table 1 and Fig. 1). These distances were selected as they have been previously found to be biologically significant^36, 38^. Next, a Gradient-based Efficient Feature Selection (GEFS) algorithm was used to evaluate all possible combinations of the 52 facial distances and select a set of facial features that maximally contributed to the overall accuracy in classifying the two sexes^37^. Then, based on the selected features, two classes (boys and girls) were created in Linear Discriminant Analysis (LDA)^39^ space. The LDA algorithm calculates the between-class-scatter and within-class-scatter using the covariance matrix and maximises the ratio of the two scatters. Finally, the training of the LDA classifier was performed using a 10-fold cross validation technique whereby data were partitioned into 10 equal sets. In each fold, nine sets were used for training the LDA classifier and one set for testing its accuracy (see Gilani *et al*.^37^ for more details). In addition, as it is conceivable that body mass index may influence facial measurements^40^, facial areas were calculated by summing the area of all triangles connected between the points in the 3D space.

**Table 1:** Summary of facial landmarks and distances measured in Study 1.

Number	Landmark	Facial distance
1	*Ft-Ft*	Forehead width
2	*Ex-Ex*	Outer-canthal width
3	*Ex-En* (*left*)	Eye fissure length (left)
4	*Ex-En* (*right*)	Eye fissure length (right)
5	*En-En*	Inter canthal width
6	*Ex-N* (*left*)	Mid face width (left)
7	*Ex-N* (*right*)	Mid face width (right)
8	*En-N* (*left*)	Nasal root height (left)
9	*En-N* (*right*)	Nasal root height (right)
10	*Al-Al*	Nose width
11	*Sbal-Sbal*	Alar-base width
12	*Ch-Ch*	Mouth width
13	*Ch-Pg* (*left*)	Mandible height (left)
14	*Ch-Pg* (*right*)	Mandible height (right)
15	*Ex-Ch* (*left*)	Upper cheek height (left)
16	*Ex-Ch* (*right*)	Upper cheek height (right)
17	*Tr-G*	Forehead height
18	*N-Prn*	Nasal bridge length
19	*N-Sn*	Nose height
20	*N-Sto*	Upper facial height
21	*Sn-Prn*	Nasal tip protrusion
22	*Sn-Sto*	Upper lip height
23	*Sn-Ls*	Philtrum length
24	*Ls-Sto*	Upper vermillion height
25	*Sto-Li*	Lower vermillion height
26	*Sto-Pg*	Mandible height

Note. Facial landmarks were defined in Farkas^38^.

## Results

From the pairs of 26 linear and geodesic distances measured, the GEFS selected 11 features which, together, maximally differentiated between the boys and girls. These distances include three linear distances (alar-base width, nose height and upper lip height) and eight geodesic distances (outer-canthal width, forehead width, forehead height, right upper cheek height, nasal tip protrusion, nose height, upper lip height, and nasal bridge length; see Table 2 for descriptive statistics). Based on these distances, the sex of the faces were classified with an accuracy of 98.3% for boys and 97.8% for girls, based on multiple classification runs.

**Table 2:** Descriptive statistics for the sexually dimorphic facial distances and facial area for each sex for Study 1.

Facial variables	Boys (*n* = 48)		Girls (*n* = 53)	
	*M*	*SD*	*M*	*SD*
Linear features (mm)				
Alar-base width^a^	15.4	1.95	14.1	1.76
Nose height^a^	44.4	4.36	40.9	4.86
Upper lip height^a^	20.3	2.52	17.7	2.15
Geodesic features (mm)				
Outer-canthal width^a^	106.7	8.48	100.5	8.09
Forehead height^a^	45.6	8.62	50.5	6.97
Forehead width	152.9	10.41	149.8	12.03
Right upper cheek height	66.3	4.97	65.4	4.48
Nasal tip protrusion	17.1	2.79	17.0	2.32
Nose height^a^	53.5	5.87	49.7	6.12
Upper lip height	24.6	3.29	20.2	8.48
Nasal bridge length	35.3	4.96	34.8	4.89
Facial area (mm^2^)	27227	4016	26563	3769

Note. ^a^There was a significant difference between typically-developing boys and girls at a Bonferroni-adjusted alpha level of 0.0045.

All facial variables were normally distributed and facial area did not significantly differ between boys and girls, *t*(99) = 0.86, *p* = 0.40 (see Table 2). Further analyses were conducted to compare the differences in the 11 features identified by GEFS between boys and girls with a Bonferroni-adjusted alpha level of 0.0045. Given that facial morphology varies with age^38, 41^, an age factor was added by dividing the total sample into the ‘younger’ and ‘older’ groups based on the median age of 8.08 years. Between-subjects ANOVAs with a 2 (sex: boys *vs*. girls) by 2 (age: younger *vs*. older) design indicated that all three linear features were significantly larger in boys compared to girls, which were alar-base width, *F*(1, 97) = 15.1, *p* < 0.001, ${\eta }_{p}^{2}$ = 0.14, nose height, *F*(1, 97) = 23.5, *p* < 0.001, ${\eta }_{p}^{2}$ = 0.20, and upper lip height, *F*(1, 97) = 31.2, *p* < 0.001, ${\eta }_{p}^{2}$ = 0.24. Three of the eight geodesic distances were significantly different between the two sexes. Boys presented larger geodesic outer-canthal width compared to girls, *F*(1, 97) = 20.4, *p* < 0.001, ${\eta }_{p}^{2}$ = 0.17, and larger geodesic nose height, *F*(1, 97) = 16.9, *p* < 0.001, ${\eta }_{p}^{2}$ = 0.15. Geodesic forehead height was significantly larger in girls compared to boys, *F*(1, 97) = 9.6, *p* = 0.003, ${\eta }_{p}^{2}$ = 0.09. Statistical outcomes for the effect of age are provided in the supplementary file (see Table S1) and there was no significant interaction between sex and age in each ANOVA.

## Study 2

In Study 1, we identified a configuration of 11 facial distances that best differentiated the faces of typically-developing boys and girls. In this study, these features were used to facilitate the comparison between facial features of children with and without ASD in two methods. First, an overall facial masculinity/femininity index was calculated for each facial image using a gender scoring algorithm^24, 37^ derived from the normative data in Study 1 (detailed description provided below). Second, the six facial features shown to be statistically different between typically-developing boys and girls were compared between the ASD and control groups for each sex.

## Method

### Participants

Fifty-four Caucasian boys with ASD and 20 Caucasian girls with ASD were recruited from the Western Australian Autism Biological Registry (WAABR), which is an ongoing study of children with ASD and their families taking place at the Telethon Kids Institute in Perth, Western Australia. In Western Australia, a diagnosis of ASD involves an assessment by a multidisciplinary team comprising a paediatrician, psychologist and speech pathologist. A diagnosis is confirmed only when the team reaches a consensus. At the time of facial photography, all participants had their diagnoses confirmed with the Autism Diagnostic Observation Schedule-Generic, which is described below^42^.

All control children in this study were Caucasian and recruited as described in Study 1. Each boy with ASD was individually matched with one typically-developing boy on chronological age (within 12 months of age; *N* = 54). Given the comparatively small sample size of girls with ASD, each girl with ASD was matched with three typically-developing girls on age (*N* = 60). To facilitate this matching, facial images of 26 control boys and 41 control girls were used in both Study 1 and 2, with the images of a further 28 control boys and 19 control girls used for Study 2 only. There was no statistical difference in age between autistic boys and control boys (ASD: *M* = 8.51 years, *SD* = 2.22, range = 4.14–12.52 years; control: *M* = 8.72 years, *SD* = 2.78; range = 3.01–12.52 years; *t*(106) = 0.44, *p* = 0.66) or between autistic girls and control girls (ASD: *M* = 7.90 years, *SD* = 2.48, range = 4.54–11.23 years; control: *M* = 8.06 years, *SD* = 2.09, range = 4.24–11.78 years; *t*(78) = 0.29, *p* = 0.77). Participants’ characteristics are presented in Table 3.

**Table 3:** Means (standard deviations) of participants’ characteristics and facial variables for Study 2.

	Boys		Girls	
	ASD (*n* = 54)	Control (*n* = 54)	ASD (*n* = 20)	Control (*n* = 60)
**Characteristics**				
Age (years)	8.51 (2.22)	8.72 (2.78)	7.90 (2.48)	8.06 (2.09)
ADOS-G				
Total	6.06 (2.17)	—	5.65 (1.79)	—
Social Affect	6.81 (2.13)	—	6.30 (1.81)	—
RBB	4.63 (2.30)	—	4.85 (3.07)	—
**Facial variables**				
Facial area (mm^2^)	27323 (3394)	27197 (4343)	27685 (3090)	26550 (3763)
Gender scores	5.53 (2.69)	7.20 (3.22)	13.19 (2.56)	16.43 (1.85)
Linear features (mm)				
Alar-base width^a^	16.8 (1.75)	15.5 (1.54)	16.1 (1.54)	14.1 (1.94)
Nose height^a^	43.9 (4.55)	41.2 (5.22)	43.7 (4.83)	40.0 (5.01)
Upper lip height^a^	23.6 (2.83)	21.3 (2.62)	20.1 (1.98)	17.2 (2.10)
Geodesic features (mm)				
Outer-canthal width^a^	108.1 (7.27)	103.7 (8.09)	106.9 (6.80)	100.3 (7.68)
Forehead height	43.3 (6.67)	47.1 (8.07)	52.2 (7.89)	50.6 (8.71)
Nose height^a^	53.5 (5.22)	50.5 (6.66)	53.7 (5.94)	48.1 (6.31)

Note. ^a^This feature was significantly larger in the autistic group than the control group in each sex at a Bonferroni-adjusted alpha level of 0.0071.

### Autism Diagnostic Observation Schedule-Generic (ADOS-G)

The ADOS-G is a semi-structured, standardised assessment of communication, social interaction, play and imagination. Children with ASD were administered the ADOS-G by a trained assessor at the Telethon Kids Institute^42^. Three continuous scores were derived from the ADOS-G data according to published guidelines^43, 44^: Total, Social Affect, and Restricted, Repetitive Behaviours (RRB) scores. These calibrated ADOS-G scores are less influenced by individual characteristics such as age, language abilities and IQ, and thus facilitate comparisons between children of varying developmental levels who have been administered different ADOS-G modules.

### 3D facial photography

The 3dMDface system and imaging procedure described in Study 1 were also used in this study to obtain 3D facial images of children with and without ASD.

### Gender scoring algorithm

**Figure 2:**
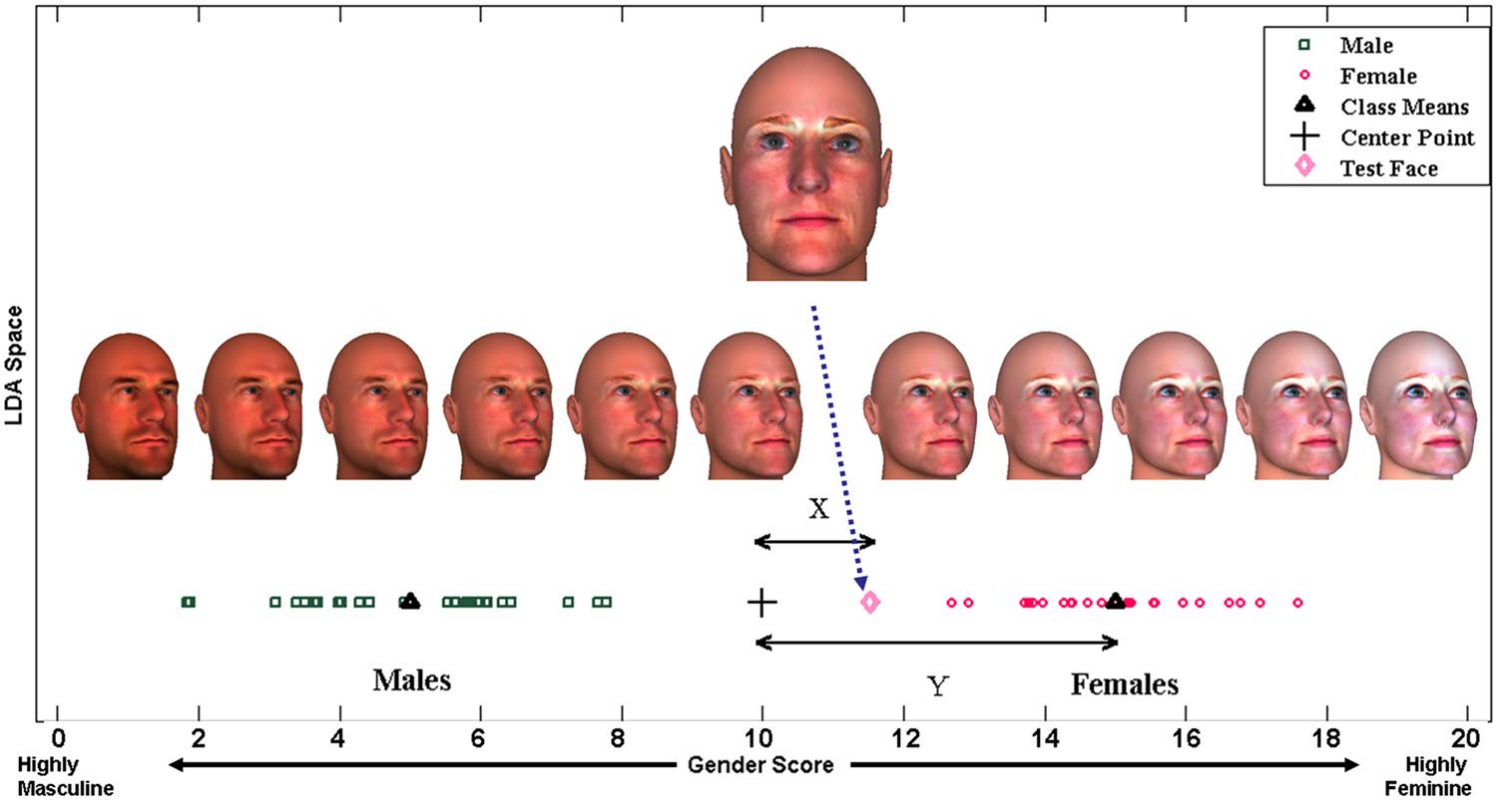
Creation of the ‘gender score’ for each face. The 11 selected features of each 3D face in Study 1 were projected in the LDA space which separates the two classes of males and females. We found the mean of both classes and the centre point between these means in the LDA space. These are shown in Fig. 2 as black triangles and a black cross. The 11 selected features of each face in Study 2 were then projected in the LDA space. The algorithm calculated the distance between the test face and the centre of the mean of the two classes (i.e., the black cross) and ascribed a ‘gender score’ as G = (1 − X)/2Y, scaled between 0 and 20. The further this test face is from the centre, the higher the masculinity or femininity. In this particular example, the test face (i.e., the pink diamond) lies between the centre point and the mean for females, which generated a ‘gender score’ that represents low femininity. The synthetic faces shown in the figure depict the varying masculinity of the same identity.

The gender scoring procedure involves three steps^37^. First, the 11 facial features identified to be sexually dimorphic in typically developing boys and girls were projected in the LDA space. Based on these features, the LDA space creates two classes, boys and girls. The mean of each class and the centre point between these means in the LDA space were calculated. In the second step, we annotated 13 landmarks on each 3D image to obtain measurements of the 11 sexually dimorphic facial features in autistic and non-autistic children included in Study 2. Each of these faces will be referred to as a ‘test face’ in the description of the algorithm. In the final step, the 11 features of each test face were entered into the LDA space where the deviation between the test face and the centre of the mean of the two classes was calculated. The deviation is represented as a ‘gender score’ which ranges from 0 (indicating extreme masculinity) to 20 (indicating extreme femininity). Figure 2 provides an illustration of the gender scoring algorithm.

### Statistical analyses

Statistical analyses were conducted separately for each sex using the following procedure. First, children with and without ASD were divided into two age groups based on median ages (8.51 years for boys and 7.93 years for girls). Then, for each sex, 2 (diagnosis: autistic *vs*. non-autistic) by 2 (age: younger *vs*. older) ANOVAs were conducted to compare the ASD and control samples on the gender scores and the six facial features shown to be statistically different between typically-developing boys and girls in Study 1 (i.e., linear: alar-base width, nose height, upper lip height, and geodesic: outer-canthal width, forehead height, and nose height). A Bonferroni-adjusted alpha level of 0.0071 was used to determine statistical significance for each of these comparisons. Partial correlation analyses were used to examine the relationship between gender scores and the three ADOS-G scores while controlling for the effect of age.

### Data availability

The datasets generated and analysed during the current study are available from the corresponding author on reasonable request.

### Ethical approval and consent to participate

Ethical approval for the study was obtained from the human research ethics committees at the University of Western Australia (RA/4/1/6668) and Princess Margaret Hospital for Children (EP/1488). Informed consents to participate and publish were obtained from parents or legal guardians. All participants were tested in accordance with the guidelines provided by the human research ethics committees at the University of Western Australia and Princess Margaret Hospital for Children.

## Results

Preliminary analyses revealed that all variables of interest were normally distributed and there was no significant difference in facial area between the ASD and control groups in each sex (boys: *t*(106) = 0.17, *p* = 0.87; girls: *t*(78) = 1.22, *p* = 0.23). Table 3 presents the descriptive statistics for the ASD and control groups in terms of gender scores, the six facial distances and facial area for each sex. Further statistical outcomes for the effects of age are summarised in Tables S2 and S3. None of the interaction terms in the ANOVAs were statistically significant.

ANOVA revealed that the autistic boys had significantly lower gender scores for their faces (i.e., more masculine) when compared to the control boys, *F*(1, 104) = 18.8, *p* < 0.001, ${\eta }_{p}^{2}\,$ = 0.15. Furthermore, five of the six facial distances were significantly larger in autistic boys than those of control boys. Autistic boys showed larger linear alar-base width, *F*(1, 104) = 18.9, *p* < 0.001, ${\eta }_{p}^{2}$ = 0.15, linear nose height, *F*(1, 104) = 16.2, *p* < 0.001, ${\eta }_{p}^{2}$ = 0.14, linear upper lip height, *F*(1, 104) = 22.4, *p* < 0.001, ${\eta }_{p}^{2}$ = 0.18, geodesic outer-canthal width, *F*(1, 104) = 15.6, *p* < 0.001, ${\eta }_{p}^{2}$ = 0.13, and geodesic nose height, *F*(1, 104) = 14.5, *p* < 0.001, ${\eta }_{p}^{2}$ = 0.12. The difference in geodesic forehead height between boys with and without ASD did not reach statistical significance with the Bonferroni correction (*p* = 0.02).

For girls, ANOVA showed that gender scores were significantly lower (i.e., less feminine) for the ASD group compared to the control group, *F*(1, 76) = 51.4, *p* < 0.001, ${\eta }_{p}^{2}$ = 0.40. Moreover, five of the six facial distances were significantly larger in autistic girls compared to control girls. In particular, autistic girls presented with larger linear alar-base width, *F*(1, 76) = 18.9, *p* < 0.001, ${\eta }_{p}^{2}$ = 0.20, linear nose height, *F*(1, 76) = 11.3, *p* = 0.001, ${\eta }_{p}^{2}$ = 0.13, linear upper lip height, *F*(1, 76) = 30.0, *p* < 0.001, ${\eta }_{p}^{2}$ = 0.28, geodesic outer-canthal width, *F*(1, 76) = 12.9, *p* = 0.001, ${\eta }_{p}^{2}$ = 0.15, and geodesic nose height, *F*(1, 76) = 17.6, *p* < 0.001, ${\eta }_{p}^{2}$ = 0.19. Geodesic forehead height was comparable for girls with and without ASD (*p* = 0.47).

**Figure 3:**
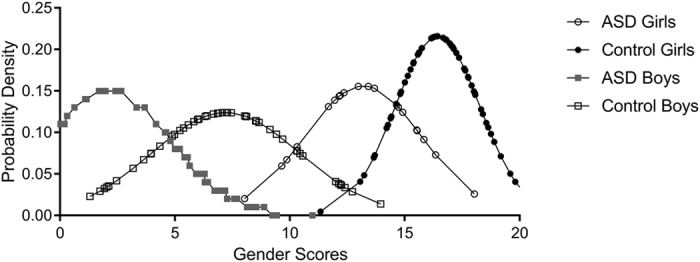
Probability density function displaying the distribution of gender scores for ASD girls (unfilled circles), control girls (filled circles), ASD boys (filled squares), and control boys (unfilled squares).

The distributions of the gender scores for the four groups of children included in this study are presented in Fig. 3. There is a clear leftward (more masculine) shift in the distributions for the autistic girls and boys compared to their typically developing same-sex counterparts.

As described earlier, to maximise the size of the ASD and control samples in Study 2, some of the typically-developing children used in Study 1 were also included in the control samples for Study 2. To check that these overlapping children did not unduly influence outcomes, we conducted further within-sex analyses comparing the gender scores and the six facial features between the ASD group and a control group comprised of children who did not participate in Study 1 (see Tables S4 and S5 for a summary of the statistical outcomes). For boys (54 autistic and 28 control), all comparisons were replicated in these additional analyses except for linear upper lip height where a trend towards statistical significance was observed (*p* = 0.068). For girls (20 autistic and 19 control), all results were reproduced except for linear nose height (*p* = 0.012) and geodesic outer-canthal width (*p* = 0.013). Thus despite lower power, these additional analyses essentially confirm the outcomes reported for the full control samples.

### Gender scores and ASD symptom severity

For the autistic boys, partial correlation analyses revealed significant negative correlations of the gender scores with both the ADOS-G total scores (*r* = −0.57, *p* < 0.001) and the Social Affect scores (*r* = −0.62, *p* < 0.001), indicating that autistic boys with more masculine overall facial morphology had more severe ASD presentations relating to social communication. However, there was no significant correlation between gender scores and RRB scores among autistic boys (*r* = 0.03, *p* = 0.84).

**Figure 4:**
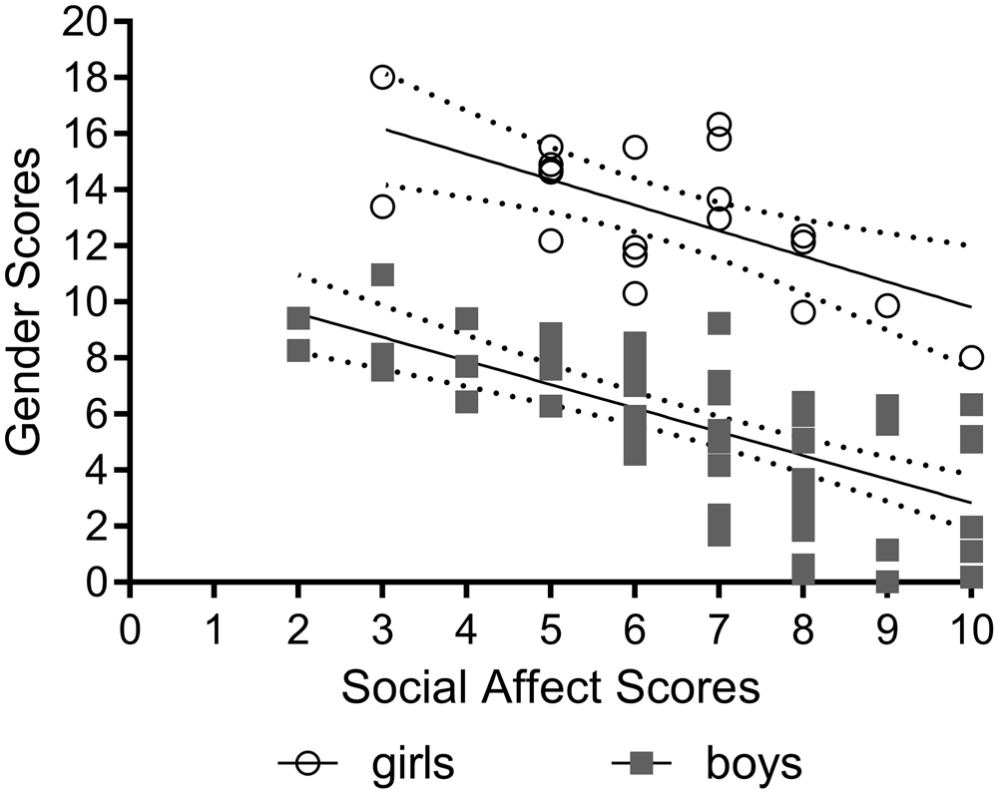
Scatterplot and trend lines showing the relationship between ADOS-G derived Social Affect scores and facial gender scores for autistic girls (unfilled circles) and boys (filled squares). Dotted lines indicate 95% Confidence Intervals for the gender scores. Facial gender scores range from extremely masculine (score of 0) to extremely feminine (score of 20).

Among girls with ASD, the partial correlations showed a significant negative correlation of gender scores with the Social Affect scores (*r* = −0.71, *p* < 0.001), indicating that autistic girls with less feminine facial structure presented with more pronounced difficulties in social communication. There was no significant correlation between gender scores and either ADOS-G total scores (*r* = −0.40, *p* = 0.09) or RRB scores (*r* = 0.41, *p* = 0.08). Figure 4 presents a scatterplot demonstrating the relationships between gender scores and Social Affect scores for boys and girls with ASD.

The relationship between gender scores and Social Affect scores for each sex was further examined using a hierarchical multiple regression analysis. As age correlated with gender scores for boys (age: *r* = −0.58, *p* < 0.001) and for girls (age: *r* = −0.78, *p* < 0.001), we entered the age variable in the first block. Gender scores were then added in the second block. For boys, gender scores were associated with the Social Affect scores (B = −0.57, p < 0.001) and their inclusion significantly improved the regression model, accounting for an additional 31.9% of the variation in Social Affect scores, *F*(1, 51) = 31.9, *p* < 0.001, for change in R^2^. Likewise, for girls, gender scores were related to Social Affect scores (B = −0.77, p = 0.001), with gender scores accounting for an additional 47.2% of the variation in Social Affect scores, *F*(1, 17) = 17.7, *p* = 0.001, for change in R^2^.

## General Discussion

The current study employed a two-step approach, first determining a set of facial features that could distinguish faces of boys and girls. These features were then used to investigate the hypermasculinisation and androgyny accounts by comparing children with and without ASD on facial masculinity/femininity. The present findings provide support for the hypermasculinisation account in which increased facial masculinity was observed in the overall facial structure and in individual features of autistic boys and girls in comparison to typically-developing controls.

Research to date, including the current study, indicate a stable pattern of results for females in which hypermasculinised facial appearance has been reported in prepubescent girls with ASD (in the current study) and in women with ASD^28^ compared to their typically-developing counterparts. However, the association between facial masculinity and autistic behaviours has been less consistent for males. While the current study found clear evidence for hypermasculinised facial features in boys with ASD, Bejerot *et al*.^28^ did not observe a difference in the facial features of autistic and non-autistic men. We raise two possibilities for the discrepant findings between the two studies.

First, there were considerable differences between studies in the methodologies used to examine facial features. Bejerot *et al*.^28^ took two-dimensional facial photographs of autistic and non-autistic adults, and had eight naïve observers rate each face for ‘gender coherence’, whereas the current study used landmark data from 3D images of autistic and non-autistic children to create objective measurements of sexually dimorphic facial features. Although existing evidence suggests that the subjective ratings of facial masculinity/femininity and the combined use of linear and geodesic measurements are positively correlated^36^, the study undertaken by Bejerot *et al*.^28^ did not directly examine masculinity/femininity. The authors interpreted low gender coherence ratings as being less feminine for females and less masculine for males, but the connotation of “gender coherence” is arguably ambiguous, that is, a less gender typical face could indicate an extremely high or an extremely low degree of masculinity/femininity. Second, the current study employed 3D photogrammetry which measures facial shape with submillimetre accuracy. It is possible that this high measurement precision identified more subtle sexually dimorphic effects that are unable to be observed through subjective ratings. Bejerot *et al*. did report subjective ratings of increased masculinity (or androgyny) in adult females with ASD, which suggests the possibility that increased facial masculinity in males with ASD may be a more subtle effect than that observed in females with ASD.

Nevertheless, despite using similar methodology as the one described in Gilani *et al*.^26^, the outcomes of the current study run counter to the findings reported in Gilani *et al*.^26^ in which more androgynous features were found in neurotypical adults with higher levels of autistic traits. An important difference between the two studies is that the current study investigated pre-pubertal children, whereas Gilani *et al*.^26^ (and also Bejerot *et al*.^28^) examined post-pubertal adults. The surge of serum testosterone concentrations during puberty is 20–30-fold higher in males compared with females^45^, and previous studies of general population samples have found that salivary testosterone levels of 12–18-year-olds are related to sexually dimorphic facial features^31^. While recent evidence suggests that prenatal testosterone exposure has a greater influence than postnatal testosterone on facial morphology in adulthood^24^, the influence of pubertal testosterone cannot be ruled out. One further possibility for the discrepant findings relates to the differences in how Gilani *et al*.^26^ and the current study measured facial distances—Gilani *et al*.^26^ measured linear distances between two facial landmarks while the current study included both linear and geodesic distances. The use of both linear and geodesic distances provides considerably higher measurement precision. Furthermore, eight of 11 distances that were identified as sexually dimorphic in Study 1 were geodesic distances. Future studies examining both linear and geodesic distances in adults with ASD will build on the findings presented here.

An important secondary finding of this study was the relationship between facial masculinity and ASD symptom severity in prepubescent boys and girls. For both sexes, an association was identified between hypermasculinised facial features (indicated by lower gender scores) and social-communication difficulties but not restricted interests and repetitive behaviours, assessed using the ADOS-G. Consistent with the current study, Obafemi-Ajayi *et al*.^35^ observed that autistic boys with more profound social-communication difficulties were characterised by facial features such as increased facial height and mouth width, and decreased mid-face height. However, in contrast to the present study, Obafemi-Ajayi *et al*.^35^ also reported an association between facial morphology and the extent of restricted interests and repetitive behaviours. One possible reason for the difference in findings relates to the difference in orientation of the two studies. Obafemi-Ajayi *et al*.^35^ focused on identifying a set of facial features in boys with ASD that deviated from those of typically-developing boys, whereas the current study framed hypotheses around the possible effects of prenatal testosterone on the levels of facial masculinisation in these two groups.

Taken together, these findings suggest that while facial phenotype specific to each behavioural domain of ASD exists, the underlying biological factors driving the development of each facial phenotype may differ. The current findings raise the possibility that prenatal testosterone has an effect on the development of atypical social-communication behaviours but exerts little influence on the restricted and repetitive behaviours in both boys and girls with ASD. Notably, ASD incorporates a high degree of phenotypic heterogeneity, and large population-based twin studies^46^ have found that the behavioural domains that characterise the condition have been found to be largely independent. The outcomes of the current study further highlight the importance of investigating aetiological factors in relation to the behavioural dimensions of ASD, rather than solely the categorical diagnostic entity^47^.

The 3D photogrammetry, measurements of linear and geodesic facial distances, two-phase validation protocol, individually-matched control samples and the inclusion of both sexes in the case-control study are the main strengths of the current study. However, we also acknowledge two limitations of our design. First, as both sample sizes in Study 1 and 2 are small, this limits our ability to generalise the current findings to the wider population. Nevertheless, sexual dimorphism in the three linear features have been previously reported in other studies^41, 48^. To the best of our knowledge, comparative data for geodesic measurements are not available in typically-developing children. Future research could perform the gender classification analyses (described in Study 1) using a larger sample to further examine sexually dimorphic facial features in the prepubescent population. Second, autistic-like traits were not assessed in the typically developing control group, thus it is possible that some control boys and girls may have had subclinical levels of autistic-like traits. However, since the gender scores and five of the six sexually dimorphic facial features differed significantly between the ASD and control groups for each sex, we do not believe the power of the current study was substantially compromised by the possible inclusion of controls with some level of autistic traits.

Given the existing evidence for the link between prenatal testosterone and facial masculinisation^24^ and the current findings on the association between facial masculinisation and ASD, it appears that prenatal testosterone may play a part in both ASD-related behaviours and morphological features. Unfortunately, data on prenatal testosterone were not available for the current samples, which limits our ability to draw firm conclusions on the hypothesised relationships. Further investigation that tracks longitudinal links between early testosterone exposure, postnatal facial morphology and the autism phenotype will provide more direct tests of the hypothesised relationships. As well as recording testosterone levels in either amniotic fluid or umbilical cord blood, it would be informative to assess peak testosterone levels in early infancy. Because the brain continues to develop after birth^49^, several studies have considered the effects of the peak in testosterone production around one to three months after birth (known as the mini-puberty period) on the extent of autistic traits later in childhood^6, 50, 51^. One of these studies reported a positive association between salivary testosterone in 3-month-old infants and autistic-like behaviours when they were 18 months old^51^ whereas the other two studies identified no such relationship^6, 50^. Determining whether this early surge in testosterone has any effect on either autistic traits or facial morphology would augment research on prenatal testosterone and its effects.

Investigations into the facial structure of individuals of ASD have the potential to reveal greater insights into the biological pathways leading to autism. Facial phenotypes are widely used in the clinical diagnosis of numerous neurodevelopmental and other disorders, and provide an important research tool for identifying rare diseases and links between genotype and phenotype. The current study adopted an hypothesis-driven approach to investigate the influence of one potential etiological factor on facial phenotype—prenatal testosterone exposure—as well as links between facial and behavioural phenotypes. However, we also see merit in adopting an hypothesis-free approach in examining differences between the ASD and typically-developing populations. Larger sample sizes will be critical to achieving this aim and understanding more about the etiological pathways underpinning ASD.

This is the first study to examine facial masculinity in prepubescent samples of children with ASD using 3D imaging technique and measurement of objective indices. Boys and girls with ASD were found to have more masculine facial features compared to age-matched typically developing controls, and increased facial masculinity was related to greater severity of social-communication behaviours. While the current evidence for the association between ASD and prenatal testosterone exposure is mixed, the present study extends on a recent finding linking elevated prenatal testosterone and increased facial masculinity^24^ and provides further evidence for the hypermasculinisation account of ASD.

## Electronic supplementary material

Supplementary information 
