# Supplementary material for: Hypermasculinised facial morphology in boys and girls with Autism Spectrum Disorder and its association with symptomatology
Source: Sci Rep. 2017 Aug 24;7:9348. doi: 10.1038/s41598-017-09939-y (PMC5570931; doi:10.1038/s41598-017-09939-y)
Supplement: Supplementary file 1 — Supplementary information [file 41598_2017_9939_MOESM1_ESM.pdf]

Hypermasculinised facial morphology in boys and girls with Autism Spectrum Disorder and its association with symptomatology

Diana Weiting Tan[1,2,†,*], Syed Zulqarnain Gilani[3, †], Murray T. Maybery[1], Ajmal Mian[3], Anna Hunt[2], Mark Walters[4], and Andrew J.O. Whitehouse[2]

[1]Neurocognitive Developmental Unit, School of Psychological Science, University of Western Australia, Perth, 6009, Western Australia, Australia

[2]Telethon Kids Institute, University of Western Australia, Perth, 6008, Western Australia, Australia

[3]School of Computer Science and Software Engineering, University of Western Australia, Perth, 6009, Western Australia, Australia

[4]Craniomaxillofacial Department, Princess Margaret Hospital for Children, Perth, 6008, Western Australia, Australia

[†]These authors contributed equally to this work.

[*]diana.tan@ uwa.edu.au

Supplementary Tables

Table S1

Means (standard deviations) and statistical outcomes for the effects of age on each facial feature for the younger (< 8.08 years) and older (> 8.08 years) children in Study 1.

	Boys (n = 48)		Girls (n = 53)		Age effect		
Facial variables	Younger	Older	Younger	Older	F(1, 97)	p	η_p^2
Linear features (mm)							
Alar-base width	15.0 (1.71)	15.9 (2.08)	13.2 (1.52)	15.0 (1.54)	16.5	< .001	.15
Nose height	42.0 (3.82)	46.7 (3.61)	37.6 (3.66)	44.0 (3.68)	56.3	< .001	.37
Upper lip height	19.8 (2.58)	20.8 (2.40)	17.5 (2.26)	18.0 (2.05)	*ns*		
Geodesic features (mm)							
Outer-canthal width	102.2 (8.02)	111.2 (6.34)	96.0 (6.05)	104.9 (7.45)	41.1	< .001	.30
Forehead height	45.1 (6.67)	46.1 (10.33)	48.9 (7.21)	51.9 (6.53)	*ns*		
Forehead width	148.8 (9.92)	156.9 (9.41)	145.9 (10.3)	153.5 (12.6)	13.8	< .001	.12
Right upper cheek height	63.8 (4.96)	68.8 (3.60)	63.4 (3.42)	67.2 (4.63)	27.3	< .001	.22
Nasal tip protrusion	16.2 (3.15)	18.0 (2.11)	16.1 (1.57)	17.8 (2.65)	12.1	.001	.11
Nose height	50.2 (4.80)	56.8 (4.94)	45.6 (4.15)	53.6 (5.13)	58.8	< .001	.38
Upper lip height	23.8 (3.21)	24.9 (3.35)	19.7 (2.91)	20.7 (1.53)	*ns*		
Nasal bridge length	33.0 (3.94)	37.7 (4.80)	31.6 (3.43)	37.8 (4.10)	45.2	< .001	.32

Table S2

Means (standard deviations) and statistical outcome for the effects of age on each facial feature and the gender score for the younger (< 8.53 years) and older (> 8.53 years) autistic and control boys in Study 2.

	Autistic boys (*n* = 54)		Control boys (*n* = 54)		Age effect		
Facial variables	Younger	Older	Younger	Older	*F*(1, 104)	*p*	η_p^2
Gender scores	6.7 (2.29)	4.1 (2.50)	9.5 (2.84)	5.4 (2.16)	50.4	< .001	.33
Linear features (mm)							
Alar-base width	16.3 (1.70)	17.3 (1.66)	15.1 (1.45)	15.8 (1.57)	*ns*		
Nose height	41.4 (3.11)	47.1 (4.08)	38.9 (4.85)	43.0 (4.81)	36.1	< .001	.26
Upper lip height	23.0 (3.04)	24.3 (2.38)	20.5 (2.50)	22.0 (2.57)	7.8	.006	.07
Geodesic features (mm)							
Outer-canthal width	105.3 (5.23)	111.5 (8.05)	98.9 (7.86)	107.6 (6.01)	32.6	< .001	.24
Forehead height	42.7 (6.66)	44.1 (6.74)	45.0 (7.02)	48.8 (8.56)	*ns*		
Nose height	50.4 (3.46)	57.5 (4.31)	47.3 (5.98)	53.0 (6.13)	42.4	< .001	.29

Note. These facial features were significantly different between typically-developing boys and girls in Study 1. An independent sample t-test revealed that the differences in the ages of boys in Study 1 and 2 did not reach statistical significance, $t(75.6) = 1.49$, $p = .14$.

Table S3

Means (standard deviations) and statistical outcome for the effects of age on each facial feature and the gender score for the younger (< 7.92 years) and older (> 7.92 years) autistic and control girls in Study 2.

	Autistic girls (*n* = 20)		Control girls (*n* = 60)		Age effect		
Facial variables	Younger	Older	Younger	Older	*F*(1, 76)	*p*	η_p^2
Gender scores	15.0 (1.67)	11.4 (1.96)	17.1 (1.64)	15.7 (1.82)	30.0	< .001	.28
Linear features (mm)							
Alar-base width	16.0 (1.72)	16.1 (1.41)	13.4 (1.99)	14.8 (1.64)	*ns*		
Nose height	40.4 (4.28)	47.0 (2.66)	37.6 (4.68)	42.5 (4.10)	*ns*		
Upper lip height	20.5 (2.10)	19.7 (1.86)	17.5 (2.11)	16.9 (2.07)	27.7	< .001	.27
Geodesic features (mm)							
Outer-canthal width	102.7 (5.84)	111.1 (4.95)	98.5 (7.38)	102.1 (7.66)	10.7	.002	.12
Forehead height	48.7 (8.57)	55.6 (5.56)	51.3 (10.05)	49.9 (7.24)	*ns*		
Nose height	49.1 (4.01)	58.3 (3.39)	45.0 (5.71)	51.3 (5.32)	33.7	< .001	.31

Note. These facial features were significantly different between typically-developing boys and girls in Study 1. An independent sample t-test revealed that the differences in the ages of girls in Study 1 and 2 did not reach statistical significance, $t(92.9) = 0.20$, $p = .84$.

As described in the paper, to facilitate the matching of the ASD and control groups on chronological age, facial images of 26 control boys and 41 control girls were used for both Studies 1 and 2, with the images of 28 control boys and 19 control girls used for Study 2 only (i.e., 'non-overlapping' samples). To check that the results for Study 2 were not unduly influenced by the overlapping cases, for each sex, we conducted a one-way ANCOVA comparing the gender scores and the six facial features between the ASD group and 'non-overlapping' control sample while controlling for the effect of age.

Table S4

Means (standard deviations) and statistical outcome for the effects of ASD diagnosis while controlling for the effect of age on each facial feature and the gender score for the autistic and 'non-overlapping' control boys in Study 2.

Facial variables	Autistic boys (*n* = 54)	Control boys (*n* = 28)	$F(1, 79)$	p	η_p^2
Gender scores	5.5 (2.69)	7.9 (2.91)	36.3	<.001	.32
Linear features (mm)					
Alar-base width	16.8 (1.75)	15.1 (1.18)	25.9	<.001	.25
Nose height	43.9 (4.55)	38.2 (3.65)	74.1	<.001	.48
Upper lip height	23.6 (2.83)	22.7 (2.24)	*ns*		
Geodesic features (mm)					
Outer-canthal width	108.1 (7.27)	100.6 (7.32)	41.2	<.001	.34
Forehead height	43.3 (6.67)	47.3 (7.13)	*ns*		
Nose height	53.5 (5.22)	46.9 (4.42)	82.7	<.001	.51

Table S5

Means (standard deviations) and statistical outcome for the effects of ASD diagnosis while controlling for the effect of age on each facial feature and the gender score for the autistic and 'non-overlapping' control girls in Study 2.

Facial variables	Autistic girls (n = 20)	Control girls (n = 19)	F(1, 36)	p	η_p^2
Gender scores	13.2 (2.56)	16.2 (1.48)	22.5	<.001	.39
Linear features (mm)					
Alar-base width	16.1 (1.54)	13.7 (2.12)	14.7	<.001	.29
Nose height	43.7 (4.83)	39.2 (4.99)	*ns*		
Upper lip height	20.1 (1.98)	17.8 (2.46)	10.5	.003	.23
Geodesic features (mm)					
Outer-canthal width	106.9 (6.80)	100.3 (7.55)	*ns*		
Forehead height	52.2 (7.89)	57.3 (8.64)	*ns*		
Nose height	53.7 (5.94)	47.0 (6.39)	11.3	.002	.24
